# Purification, Characterization, Identification, and Anticancer Activity of a Circular Bacteriocin From *Enterococcus thailandicus*

**DOI:** 10.3389/fbioe.2020.00450

**Published:** 2020-06-23

**Authors:** Lamiaa A. Al-Madboly, Nehal M. El-Deeb, Amal Kabbash, Manal A. Nael, Ahmed M. Kenawy, Amany E. Ragab

**Affiliations:** ^1^Department of Pharmaceutical Microbiology, Faculty of Pharmacy, Tanta University, Tanta, Egypt; ^2^Biopharmaceutical Products Research Department, Genetic Engineering and Biotechnology Research Institute, City of Scientific Research and Technological Applications (SRTA-City), Alexandria, Egypt; ^3^Department of Pharmacognosy, Faculty of Pharmacy, Tanta University, Tanta, Egypt; ^4^Department of Pharmaceutical Chemistry, Faculty of Pharmacy, Tanta University, Tanta, Egypt; ^5^Nucleic Acids Research Department, Genetic Engineering and Biotechnology Research Institute, City of Scientific Research and Technological Applications (SRTA-City), Alexandria, Egypt

**Keywords:** purification, circular, bacteriocin, anticancer, protein sequence

## Abstract

New anticancer agents are continually needed because cancerous cells continue to evolve resistance to the currently available chemotherapeutic agents. The aim of the present study was to screen, purify and characterize a hepatotoxic bacteriocin from *Enterococcus* species. The production of bacteriocin from the *Enterococcus* isolates was achieved based on their antibacterial activity against indicator reference strains. *Enterococcus* isolates showed a broad spectrum of antibacterial activity by forming inhibition zones with diameters ranged between 12 and 29 mm. The most potent bacteriocin producing strain was molecularly identified as *Enterococcus thailandicus*. The crude extracted bacteriocin was purified by cation exchange and size exclusion chromatography that resulted in 83 fractions. Among them, 18 factions were considered as bacteriocins based on their positive antibacterial effects. The anticancer effects of the purified bacteriocins were tested against HepG2 cell line. The most promising enterocin (LNS18) showed the highest anticancer effects against HepG2 cells (with 75.24% cellular inhibition percentages), with IC50 value 15.643 μM and without any significant cytotoxic effects on normal fibroblast cells (BJ ATCC® CRL-2522™). The mode of anticancer action of enterocin LNS18 against HepG2 cells could be explained by its efficacy to induce cellular ROS, decrease HepG2 CD markers and arrest cells in G0 phase. Amino acid sequence of enterocin LNS18 was determined and the deduced peptide of the structural gene showed 86 amino acids that shared 94.7% identity with enterocin NKR-5-3B from *E. faecium*. Enterocin LNS18 consisted of 6 α-helices; 5 circular and one linear. Model-template alignment constructed between enterocin LNS18 and NKR-5-3B revealed 95.31% identity. The predicted 3D homology model of LNS18, after circularization and release of 22 amino acids, showed the formation of a bond between Leu23 and Trp86 amino acid residues at the site of circularization. Furthermore, areas of positive charges were due to the presence of 6 lysine residues resulting in a net positive charge of +4 on the bacteriocin surface. Based on the above mentioned results, our characterized bacteriocin is a promising agent to target liver cancer without any significant toxic effects on normal cell lines.

## Introduction

Cancer is a major non-communicable disease that leads to morbidity and mortality throughout the entire world (Ferlay et al., 2013). It is defined as uncontrolled growth of cells that have escaped the equilibrium rates between cell division and cell death. Furthermore, tumor cells never respond to the normal regulation mechanisms within the body causing the emergence of cellular clones with new traits forming neoplasms (Hanahan and Weinberg, 2011). The use of conventional therapeutic agents, targeting the actively growing population, has showed a failure in the treatment of cancer due to the development of resistance. Therefore, the search for novel compounds or strategies with selective toxicity to cancer cells became an urgent necessity (Raguz and Yagüe, 2008). With the discovery of therapeutic peptides, scientists have turned their attention to the use of bacteriocins as a new therapeutic remedy against cancer (Kaur and Kaur, 2015).

Bacteriocins are peptides with cationic nature produced by a wide range of bacteria. They have a potent antimicrobial activity at very low concentrations (Jennsen et al., 2006). Bacteriocin producing bacteria are protected against the action of their own peptides. Bacteriocins are classified into two main categories based on their structures and physico-chemical properties. Class I bacteriocins are termed as lantibiotics due to the presence of the *meso*-lanthionine and 3-methyllanthionine residues. Lanthionine (Lan) is composed of two alanine residues crosslinked through a thioether bond linking their β-carbons; 3-methyllanthionine (MeLan) have one additional methyl moiety. Lantibiotics are post-translationally modified in two-step to introduce Lan and MeLan residues. Specific enzymes that dehydrate Serine and Threonine residues to dehydroalanine and dehydrobutyrine, respectively. The thiols from cysteines are subsequently added to the dehydroalanine and dehydrobutyrine via Michael type addition resulting in the formation of Lan and MeLan, respectively (Arnisona et al., 2013). Lantibiotics are subdivided into two groups; type A and type B that include thermostable peptides with small molecular size (<5 kDa). The molecular size of type A lantibiotics, ranges between 2 and 4 kDa with a net positive charge, is able to form a pore in the target cell membrane. Type B lantibiotics show a size ranges between 2 and 3 kDa and a net charge either negative or neutral. They usually interfere with the enzymatic reactions responsible for cell wall biosynthesis. Mersacidin is an example of class I bacteriocin that is produced by *Bacillus* spp. (Sahl and Bierbaum, 1998).

The second class of bacteriocins termed non-lantibiotics includes non-modified peptides or those with some minor modifications like end-to-end circularization or disulfide bridge. This class is characterized by expanded inhibitory spectrum. The non-lantibiotics are further categorized into (i) subclass IIa includes pediocin-like bacteriocins, which are characterized by the presence of a conserved *N*-terminal sequence “YGNGVXC,” (ii) subclass IIb consists of two-peptide bacteriocins, (iii) subclass IIc includes cyclic bacteriocins, and (iv) subclass IId consists of non-pediocin-like single-peptide bacteriocins (Kaur and Kaur, 2015; Tymoszewska et al., 2017).

Some bacteriocins have shown selectivity toward cancer cells with unknown mechanism of action. There are some factors that may be attributed to the selectivity of bacteriocins toward cancer cells rather than normal cells. Firstly, cancer cells have negatively charged surface due to the presence of substantial amounts of anionic phosphatidyl serine, gangliosides, heparin sulfates and *O*-glycosylated mucins. The outer surface of non-cancer cells consists of neutral phospholipids including sphingomyelins and phosphatidyl choline whereas the inner surface contains amino-phospholipids (Riedl et al., 2011). Accordingly, the cationic peptides of bacteriocins have a high affinity toward the negatively charged surface of the cancer cells rather than non-cancer cells (Kaur and Kaur, 2015). Secondly, the high fluidity of the tumor cell membrane results in destabilization, which can explain the selective cytotoxicity toward cancer cells compared to non-cancer cells. Lastly, the presence of a large number of microvilli in the cancer cell membrane facilitates the binding and uptake of bacteriocin molecules (Kaur and Kaur, 2015).

Lactic acid bacteria (LAB) is one of the most important sources of bacteriocins particularly enterococci which, have taken a great attention as an important group of LAB with various benefits rather than being known as common pathogens (Franz et al., 2011). The present study aimed to extract, purify, characterize, and identify a bacteriocin from enterococci and to evaluate its anticancer activity.

## Materials and Methods

### Reference Strains

Four reference strains were used including; *Staphylococcus aureus* ATCC 29213, *Streptococcus thermophilus* ATCC 19258, *E. coli* ATCC 8739, and *Pseudomonas aeruginosa* ATCC 27950.

### Cell Lines

HepG2 (ATCC® HB-8065™) and human fibroblast cells (BJ ATCC® CRL-2522™) were obtained from the laboratory of the Tissue Culture Department of the Holding Company for Biological Products and Vaccines (VACSERA), Cairo, Egypt.

### Enterococci Isolation and Screening for Bacteriocin Production

Using bile esculin agar, enterococci (E_1_-E_30_) were isolated from dairy products such as fermented milk that was obtained from the Faculty of Agriculture, Tanta University, Tanta, Egypt. The isolates were identified using API-20S kit. Furthermore, agar spot method was used in the screening process for bacteriocin production from enterococci bacterial isolates based on their antibacterial activity against different Gram-positive and -negative pathogens (Todorov and Dicks, 2004; Eamanu et al., 2005). To rule out the antibacterial effects of enterococci organic acids and hydrogen peroxide, cell free supernatant (CFS) was neutralized with 1N NaOH and catalase enzyme (Sigma-Aldrich, USA, 68 U/ml). To test the sensitivity of the bacteriocins to proteolytic enzymes, proteinase K (Sigma-Aldrich, USA, 1 mg/ml) was added to the neutralized CFS (Schillinger and Lucke, 1989; Todorov and Dicks, 2004).

The most promising bacteriocin producing *Enterococcus* strain was selected for further identification using 16S rRNA gene sequencing as described by Sambrook et al. (1989) and Ausubel et al. (2002). Briefly, genomic DNA was extracted from an overnight culture of the selected isolate (E5) using GenJET Genomic DNA purification Kit (Thermo Scientific, EU Lithuania) according to the manufacturer's instructions. Identification of the target isolate was done by 16S rRNA gene sequencing using universal primers AGAGTTTGATCTGGCTCAG and TACGGACCTTGTTACGACTT. The PCR reaction mixture contained 10 pmol of each primer, 10 ng of genomic DNA, 2.5 mmol/L dNTPs, and 2.5 U of Taq polymerase in 50 ml of polymerase buffer. The reaction was run for 34 cycles at 94°C for 1 min as denaturation step, 55°C for 1 min as an annealing step, and 72°C for 10 min (extension step). The amplicon was then subjected to gel electrophoresis at 100 V for 1 h on 1% (mg/mL) agarose gel in Tris-acetate-EDTA buffer using a 1 Kb ladder for size verification, visualized by gelRed staining and viewed at 1,450 bp using the ChemiDoc™ XRS System UV transilluminator (Bio-Rad). PCR product was purified using the GenCatch™ PCR Cleanup Kit (Epoch Life Science, USA). Finally, pure PCR product was sequenced using an ABI 3730XL DNA analyzer (Applied Biosystems, USA).

### Bacteriocin Production Kinetics

The selected *Enterococcus* isolate was grown in 200 ml of De Man-Rogosa-Sharpe broth at 37°C in a shaking incubator at 150 rpm. Duplicate samples were withdrawn at 2 h intervals over 72 h. Bacterial growth was monitored over the time intervals by measuring cell density at 600 nm, then the collected samples were centrifuged, neutralized to pH 6.8, filtered and used to assess the bacteriocin activity as previously described above. All measurements were performed in triplicate and the average was calculated ± standard deviation (SD) (Todorov and Dicks, 2004).

### *Enterococcus* Bacteriocin Extraction and Purification

Bacteriocin extraction and purification were carried out using ammonium salt precipitation and column chromatography (Yang et al., 1992). Briefly, different concentrations of ammonium sulfate (10, 20, 30, 40, 50, and 60%) were added to 100 ml of the cell free supernatant (crude bacteriocin) and precipitated at 4°C. After 8 h, the precipitate was collected by centrifugation at 6,100 g for 20 min, then the collected pellet was weighed and re-suspended in acetonitrile solution. The crude extract of bacteriocin was desalted by C18 cartridge (Sep-Pak, Waters, USA), which was prewashed with acetonitrile containing 0.1% triflouroacetic acid (TFA) conditioned with 0.1% TFA, then eluted in 50% acetonitrile with 0.1% TFA (Yanagida et al., 2005). Eluted bacteriocins were lyophilized and re-suspended in 50 mM sodium phosphate buffer A, 5.7) and transferred to a cation exchange column (SP-Sepharose, GE Healthcare Life Science, USA) preconditioned with buffer A. Furthermore, a gradient of 0.8 M NaCl in buffer A was used to elute bacteriocin fractions. Collection of eluted fractions and testing against the indicator strain (*S. aureus* ATCC 29213) were performed. Active bacteriocin-containing fractions were desalted and concentrated by C18 cartridges, then eluted using 50% acetonitrile with 0.1% TFA, and freeze-dried. Active bacteriocin peptides were subsequently loaded on a size exclusion column (5 *u*m, 300 × 8 mm, ReproSil 50 SEC, Germany) integrated into HPLC system (Hitachi, Japan).

The titer of bacteriocin was determined as described by Todorov and Dicks (2004). The molecular size of the purified bacteriocin factions was determined by TRIS-tricin- PAGE gel using 15% resolving gel as described by Laemmli (1970). After electrophoresis, the gel was stained with Coomassie Brilliant Blue R-250 (Sigma, USA). A protein molecular weight marker (10–179 kDa; Prosieve, cat. no. 50552) containing nine polypeptides was used to determine the average molecular weight of separated proteins.

### Lytic Activity of the Purified Bacteriocins

To detect the lytic activity of the purified bacteriocins, a parallel zymogram TRIS-tricin-PAGE gel was prepared and run in a similar manner as previously described. Furthermore, after completion of the electrophoresis run, gel was placed onto Brain Heart Infusion (BHI) agar surface overlaid with the indicator strain (*S. aureus* ATCC 29213), then incubated overnight at 37°C (Beukes et al., 2000; Chang et al., 2013). The gel was compared with the Coomassie stained one to locate the active band, and hence determining its approximate molecular weight.

### Safety Pattern of the Purified Bacteriocins

The safety pattern of the test bacteriocins was evaluated on normal fibroblast cell line using neutral red assay. Briefly, about 100 μl of each of serially diluted bacteriocin was incubated with pre-cultured (6 × 10^4^ cell/ml) cells in 96-well plates. After 48 h of incubation, the cytotoxic effects of the fractions on the tested cells were quantified using neutral red assay method (Borenfreund and Puerner, 1985).

### Anticancer Activity of the Purified Bacteriocins

The anticancer effects of the positive bacteriocins-containing fractions were tested against HepG2 cells using neutral red assay method (Borenfreund and Puerner, 1985) as described above.

### Selectivity Index (SI)

Bacteriocin selectivity index to HepG2 cell line comparing with the normal cell line was calculated as previously mentioned by Koch et al. (2005); SI = IC50nc/IC50cc where IC50nc refers to the IC50 value of bacteriocin on normal fibroblast cells and IC50cc refers to the IC50 of bacteriocin on HepG2 cell line.

### Reactive Oxygen Species (ROS) Induction Using Bacteriocin

About 2 × 10^5^ cells/ml of HepG2 or fibroblast cells were grown in RPMI medium and seeded into a rounded bottom 96-well plate for 24 h. Following incubation, cells were tested in the presence (15.6 μM) or absence of the selected bacteriocin for 24 h. The intracellular induced ROS was assessed using the fluorescent membrane permeable probe 2,7-dichlorofluorescein diacetate (DCFH-DA) (Molecular Probes, Sigma Aldrich). The fluorescent probe was added at the end of the treatment in a final concentration of 20 μM. After incubation, the resulted fluorescence was measured using flow cytometry (Sawada et al., 1998; El-Adawi et al., 2012).

### Cell Cycle Analysis

HepG2 cells cycle pattern was checked after bacteriocin treatment using flow cytometry and according to Léonce et al. (2001). In this assay, propidium iodide (PI) can be used to discriminate the living from the dead cells and for cell cycle analysis. Cell cycle analysis is based on the stoichiometric binding of PI to intracellular DNA. At the end of treatment with the selected bacteriocin, HepG2 cells were washed and collected by trypsinization. The cells were then re-suspended in warm PBS and fixed with about 4 ml ice cold absolute ethanol. Finally, the cells were stained with 0.5 mL of warm PI solution (7 mL of PI solution consists of 0.35 mL of PI stock solution [1 mg/mL], 0.7 mL RNase A stock solution [1 mg/mL], and 6 mL of PBS) and incubated in darkness for 30 min. The samples were kept on ice until flow cytometric analysis.

### The Effect of the Test Bacteriocin on the Expression of HepG2 Cells CD Markers

This assay was carried out according to Léonce et al. (2001) and Borhani-Haghighi et al. (2015) with some modifications. At the end of HepG2 cellular treatment, the surface markers that expressed on HepG2 cells were analyzed using flowcytometry.

Aliquots of the control and bacteriocin treated cells (10^5^ cells/ml) were stained by anti-human APC-CD44, PE-CD34, and FITC-CD105 antibodies (Abcam, Cambridge, UK), for 30 min at 4°C. Finally, the samples were analyzed on a FACS Calibur machine using CELLQUEST software.

### Characterization and Identification of the Purified Enterocin LNS18

The selected purified bacteriocin that showed the highest anticancer activity was subjected for characterization. The effect of different detergents, enzyme inhibitors, pH and heat on the bacteriocin activity was evaluated. Different concentrations of detergents and surfactants including; SDS, urea, tritone-X100, tween 80, EDTA were used to test the bacteriocin sensitivity. Also, proteinase-K, α-amylase, pronase E, lysozyme, trypsin, chymotrypsin, and catalase were used at 0.1 mg/ml to check the bacteriocin sensitivity. All the above mentioned variables were tested separately on the purified bacteriocin for 1 h at 37°C.

Bacteriocin sensitivity to chloroform was tested by mixing with an equal volume of chloroform and kept at room temperature for 4 h prior to antimicrobial activity testing. The pH of the purified bacteriocin was adjusted to 2, 4, 6, 8, and 10 then kept at room temperature for 10 min, then checked for its antibacterial activity.

Bacteriocin thermal stability was checked by heating the sample at 100°C for 30 and 60 min and at 121°C for 15 min, then bacteriocin antibacterial activity for each treatment was subsequently assayed. The bacteriocin activity is defined as the reciprocal of the highest dilution at which the indicator strain was inhibited and it is expressed as activity units per milliliter AU/ml (Todorov and Dicks, 2004; Rajaram et al., 2010).

Isoelectric focusing point (pI) was carried out according to Laemmli (1970) and Chee et al. (2014).

### *N*-Terminal Amino Acids Sequencing

The *N*-terminal amino acid sequence of the most biologically active bacteriocin (enterocin LNS18) was analyzed depending on Edman degradation using a protein sequencer PPSQ-21 model (Shimadzu, Japan) (Sawa et al., 2009). Analysis of the test bacteriocin treated with BNPS-skatole (3-Bromo-3-methyl-2-(2-nitrophenylthio)-3H-indole) was also performed. This type of treatment, cleaves on the *C*-terminal side of tryptophan residues, was carried out as previously described (Himeno et al., 2015), and the resultant peptide fragment was purified by reverse-phase HPLC, as previously reported (Ishibashi et al., 2012). Edman degradation sequencing of this peptide fragment was followed.

### Molecular Mass Determination by MALDI-TOF-MS

The molecular mass of enterocin LNS18 was confirmed by mass assisted laser desorption ionization time of flight mass spectrometry (MALDI-TOF MS). The sample was prepared at a 1:1 dilution, with a matrix composed of (3 mg/mL α-cyano-4-hydroxycinnamic acid; CHCA) in 50% (v/v) acetonitrile, and 1% (v/v) formic acid before spotting on an Anchor Chip (Bruker- Daltonics, Bremen, Germany) and dried at RT then analyzed with an Autoflex III TOF/TOF mass spectrometer (Bruker-Daltonics).

### Nucleotide Sequence Analysis of the Structural Gene

To clone the gene (*enkB*) encoding for the test enterocin precursor peptide, polymerase chain reaction (PCR) and DNA sequencing were carried out using the primers reported in Himeno et al. (2015), and a cloning host strain *(E. coli* DH5α). Total DNA was extracted from *E. thailandicus*, using DNA extraction kit. Restriction endonucleases were used to digest DNA including; *Bam*HI, *Eco*RI, *Hind*III, *Kpn*I, *Spe*I, or *Xba*I (Nippon Gene, Japan), then ligation of digested DNA into a pUC18 cloning vector, which was previously treated with the corresponding restriction endonucleases and then dephosphorylated. Ligated products were used as template for PCR. To get the target gene, the degenerate primers (Bd-F1, Bd-F2, and Bd-F3) were used based on the obtained amino acid sequence as well as vector specific primers for ligation-anchored and nested PCR. In order to amplify the upstream region of the target gene, the degenerate primers (Bd-R1, Bd-R2, and Bd-R3) were used. Purification of the obtained fragments were performed using a QIAquick PCR purification kit (Qiagen, Germany), then sequenced. The amplified fragments were cloned and sequenced, and then a new set of specific primers (PB-F1 and PB-R1) was used to confirm the DNA sequence of the enterocin encoding gene and its vicinity. The resultant DNA sequence was analyzed using BLAST of NCBI (http://www.ncbi.nlm.nih.gov/).

### Computer Analysis

Searching for homology, multiple sequence alignment, and phylogenetic tree construction were done using NCBI **BLASTp** tool. (https://blast.ncbi.nlm.nih.gov/Blast.cgi?PROGRAM=blastp&PAGE_TYPE=BlastSearch&LINK_LOC=blasthome). The predicted pI of LNS18 sequence was performed using Compute pI tool: **EXPASY** (http://web.expasy.org/compute_pi). **Clustal Omega** was also used to construct multiple sequence alignments; it is available by the EMBL-EBI (European Bioinformatics Institute) online server (https://www.ebi.ac.uk/Tools/msa/clustalomega). The helical wheel output was created (http://rzlab.ucr.edu/scripts/wheel. Id: **wheel**. pl,v 1.4 2009-10-20). Predicted membrane topology was visualized using **Protter** (http://wlab.ethz.ch/protter). For prediction of secondary LNS18 structure, the fully automated protein structure homology-modeling **SWISS-MODEL** server was used.

**PyMol** software (1.8.3, Schrödinger, LLC) was used to generate the 3D Model of LNS18 bacteriocin and presented as cartoon. Net charge on the protein was calculated using protein tool; **Prot pi** (https://www.protpi.ch/Calculator/ProteinTool).

### Statistics

Values of the results correspond to the means of three independent tests ± (standard deviation SD). Statistical analysis of the data was performed using SPSS software (version 17). For testing the biochemical nature of bacteriocin, one-way analysis of variance (ANOVA) was applied to the experimental data considering a value of α < 0.05. Because *P* < α, a significant difference was found between the means of the groups. In addition, equal variances were assumed according to the *post-hoc* test.

## Results

Thirty *Enterococcus* sp. were isolated from dairy products using bile esculin agar that revealed pinpoint black colonies with blackening in the surrounding medium. Identification of enterococci at the species level was carried out using API-20S kit that revealed 10 *E. faecalis*, 5 *E. faecium*, 8 *E. durans*, 2 *E. thailandicus*, and 4 *E. avium*. Following preliminary screening for antibacterial efficacy, only 16 out of 30 (53.3%) *Enterococcus* isolates showed positive result with a broad spectrum activity against Gram-positive and -negative strains as observed from Table 1. Inhibition zone diameters ranged between 13 and 29 mm.

**Table 1:** Assessment of the antibacterial activity of *Enterococcus* sp. CFS against the indicator strains.

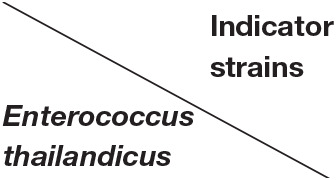	**Inhibition zone diameters in (mm) against Gram-positive and –negative indicator strains**			
	***S. aureus***	***S. thermophilus***	***E. coli***	***P. aeruginosa***
E1	10 ± 0.2	11 ± 0.28	10 ± 0.02	0
E2	22 ± 0.2	22 ± 0.64	9 ± 0.85	9 ± 0.04
E3	17 ± 0.09	17 ± 0.08	11 ± 0.11	10 ± 0.11
E5	28 ± 0.11	26 ± 0.07	16 ± 0.24	16 ± 0.35
E7	19 ± 0.5	20 ± 0.11	10 ± 0.35	9 ± 0.64
E9	29 ± 0.07	27 ± 0.11	12 ± 0.85	11 ± 0.24
E10	22 ± 0.33	20 ± 0.04	12 ± 0.35	12 ± 0.58
E11	23 ± 0.07	21 ± 0.08	12 ± 0.07	10 ± 0.11
E12	23 ± 0.45	25 ± 0.06	14 ± 0.05	13 ± 0.35
E14	20 ± 0.14	18 ± 0.17	11 ± 0.33	10 ± 0.33
E15	19 ± 0.25	14 ± 0.58	9 ± 0.11	0
E17	21 ± 0.14	23 ± 0.31	13 ± 0.25	10 ± 0.66
E19	13 ± 0.22	14 ± 0.04	15 ± 0.33	12 ± 0.17
E20	15 ± 0.68	18 ± 0.22	10 ± 0.14	9 ± 0.25
E26	22 ± 0.22	23 ± 0.35	11 ± 0.11	10 ± 0.07
E29	18 ± 0.14	15 ± 0.38	11 ± 0.58	10 ± 0.05

Neutralization of the cell free supernatant showed that only 3 isolates; E_5_, E_9_, and E_12_ retained their antibacterial activity with non-significant reduction in the inhibition zones diameters (*p* ≥ 0.05). Treatment of the later strains' CFS with catalase enzyme resulted in dramatic reduction in the inhibition zone diameters of E_9_ and E_12_. Furthermore, mixing proteinase K with the CFS of E_5_ (*E. thailandicus*) showed slight reduction in the inhibition zones (Supplementary Data 1). Identification of the selected isolate (E5) was confirmed by 16S rRNA gene sequencing and the sequence was deposited in the gene bank under an accession number of MF973085.1 (https://www.ncbi.nlm.nih.gov/nuccore/MF973085.1/). A phylogenetic tree was developed for the candidate isolate along with the sequences collected from the GenBank database, using BioEdit 7.0.5.3 and TreeViewX, for the target isolate as shown in Supplementary Data 2A.

Bacteriocin production from E5 started at the early logarithmic phase 6 h post-incubation. The production was continued until it reached the maximum titer, 12,800 AU/ml, at the beginning of the stationary phase (24 h post-incubation). Additionally, it remained stable for 72 h (Supplementary Data 2B).

**Figure 1:**
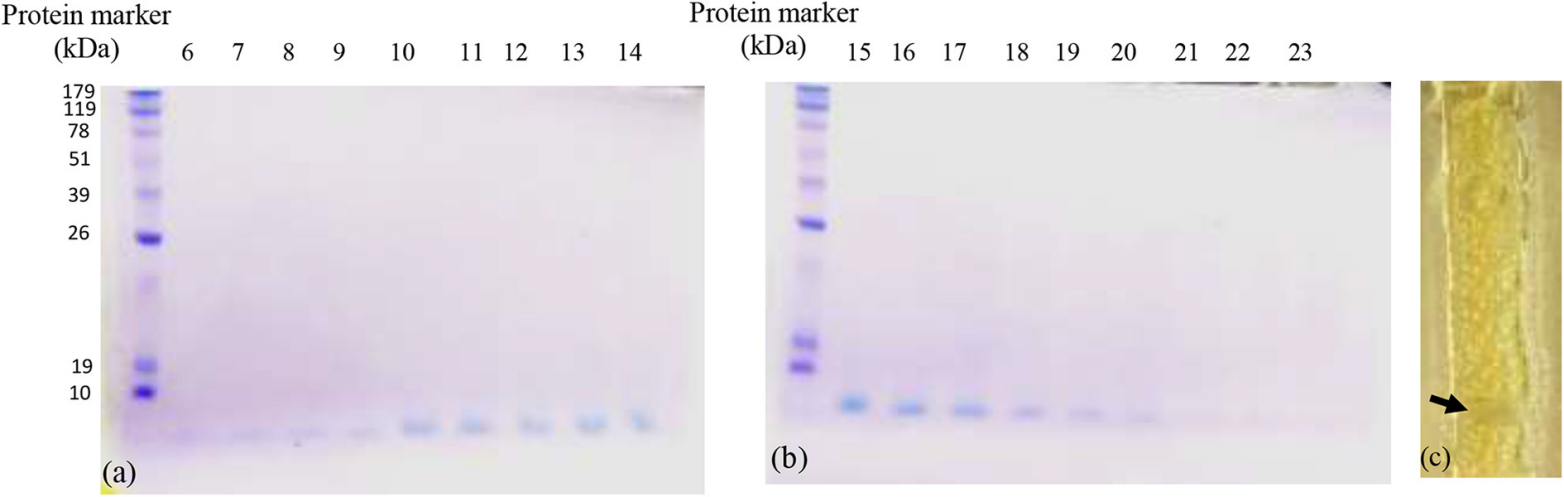
The TRIS-tricin-PAGE of 18 positive bacteriocin factions of *E. thailandicus* E5 isolate showing molecular weight below 10 kDa. Corresponding bands of fractions 6–14 **(a)**, Corresponding bands of fractions 15–23 **(b)**, zymogram of fraction E5-F12 showing growth inhibition of the indicator strain, *S. aureus* as pointed by the arrow **(c)**.

Upon purification of bacteriocin by column chromatography, 18 fractions out of 83 showed positive antibacterial activity against the indicator strain (*S. aureus*). All fractions loaded on 15% TRIS-tricin-PAGE gel showed a protein band of molecular weight <10 kDa as shown in Figures 1a,b. A representative bacteriocin band showing inhibited growth was observed in the zymogram (Figure 1c).

**Figure 2:**
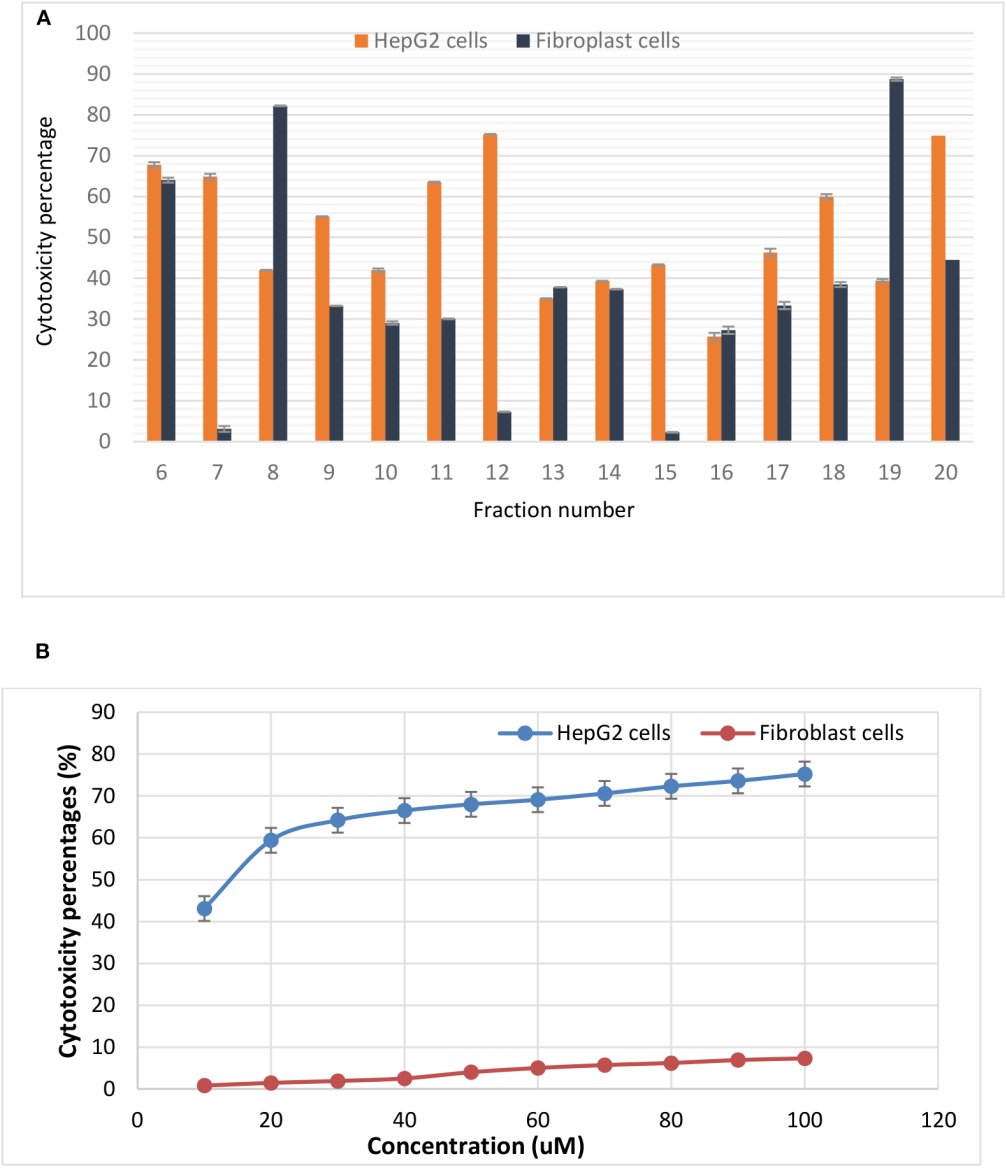
Cytotoxic effects of **(A)** various purified bacteriocins on HepG2 cell line and fibroblasts, **(B)** the IC50 values of bacteriocin from fraction 12 against HepG2 as well as the normal fibroblasts. It is more selective to HepG2 cells as compared to normal fibroblasts.

Among the tested bacteriocins, that obtained and purified from fraction 12 (E5-F12) which, showed the highest activity against HepG2 cells. Using 100 μM concentration, the inhibition percentage reached 75.24 by this bacteriocin with low toxicity percentage on the fibroblast cells (7.3). Additionally, both bacteriocins 7 and 11 showed inhibition percentages against HepG2 cells reached up to 64.8 and 63.5, respectively with toxicity percentages on the fibroblast 3.1 and 30.05, respectively (Figure 2A). On the other hand, bacteriocins from fractions 8 and 19 showed opposite behavior compared to others. Neutral red assay was used to determine the IC_50_ of the most promising bacteriocin. It revealed IC_50_ values of 15.643 and 220.345 μM against HepG2 and Fibroblast, respectively as shown in Figure 2B. Furthermore, testing bacteriocin concentrations above 100 μM showed no added cytotoxicity on HepG2 cells (data not shown). Interestingly, the calculated selectivity index was 14 indicating greater selectivity.

**Figure 3:**
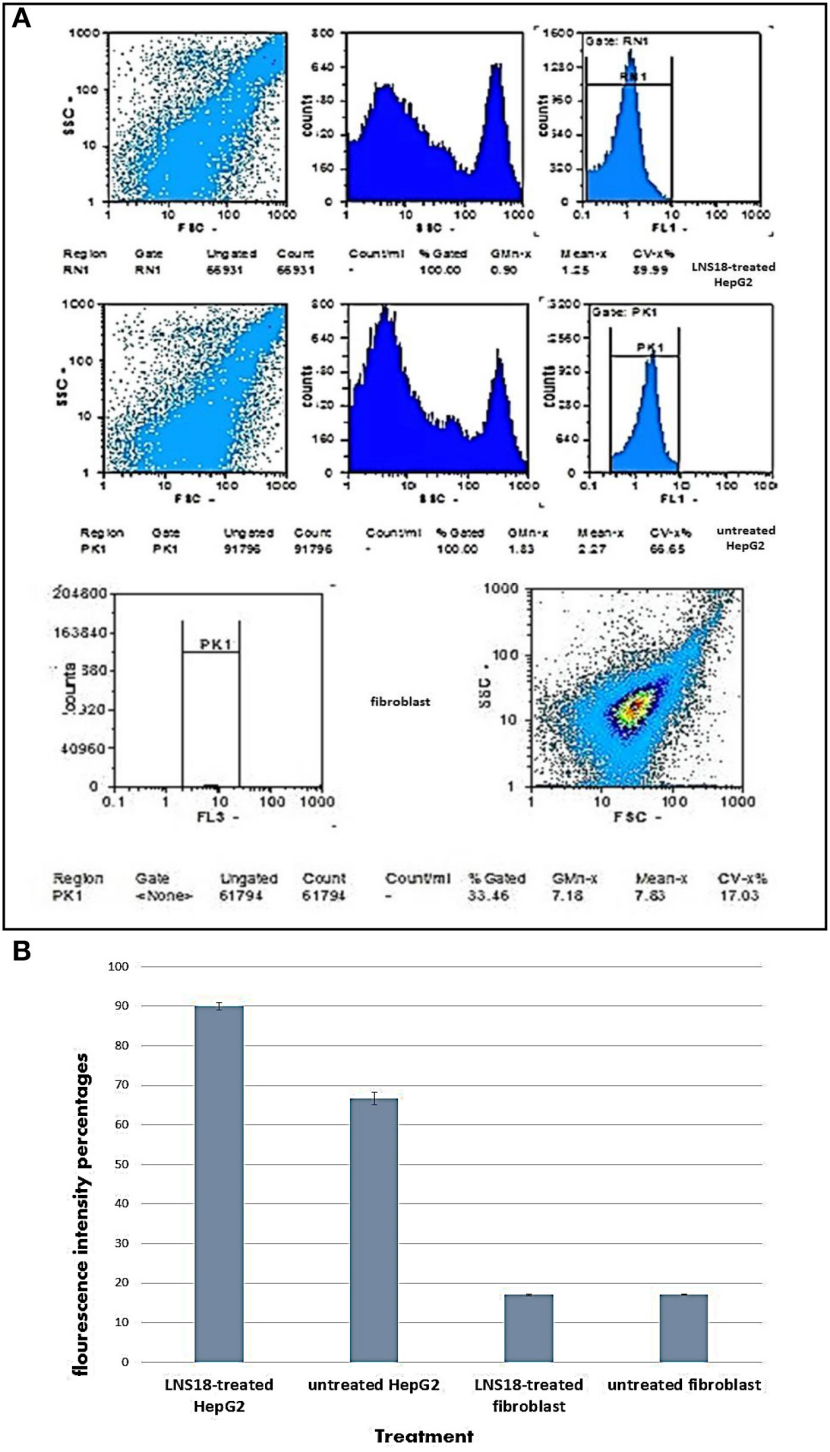
Flow cytometry output showing induction of reactive oxygen species (ROS) by the purified enterocin LNS18 in HepG2 as well as the fibroblasts **(A)**, bar chart showing increased fluorescence intensity percentage of treated HepG2 cells by 89.99% indicating induction of ROS production by the test bacteriocin **(B)**.

The levels of intracellular induced ROS after treatments were monitored by measuring the emitted relative fluorescent by flow cytometry upon oxidation of the reduced form of the fluorescent probe (DFCH). The obtained data in Figure 3 showed an increase in DCF fluorescence in case of HepG2 treated cells (89.99%) compared with the non-treated cells (66.65). On the other hand, fibroblasts recorded minimal percentage of ROS (17.03) for both treated and untreated cells.

**Figure 4:**
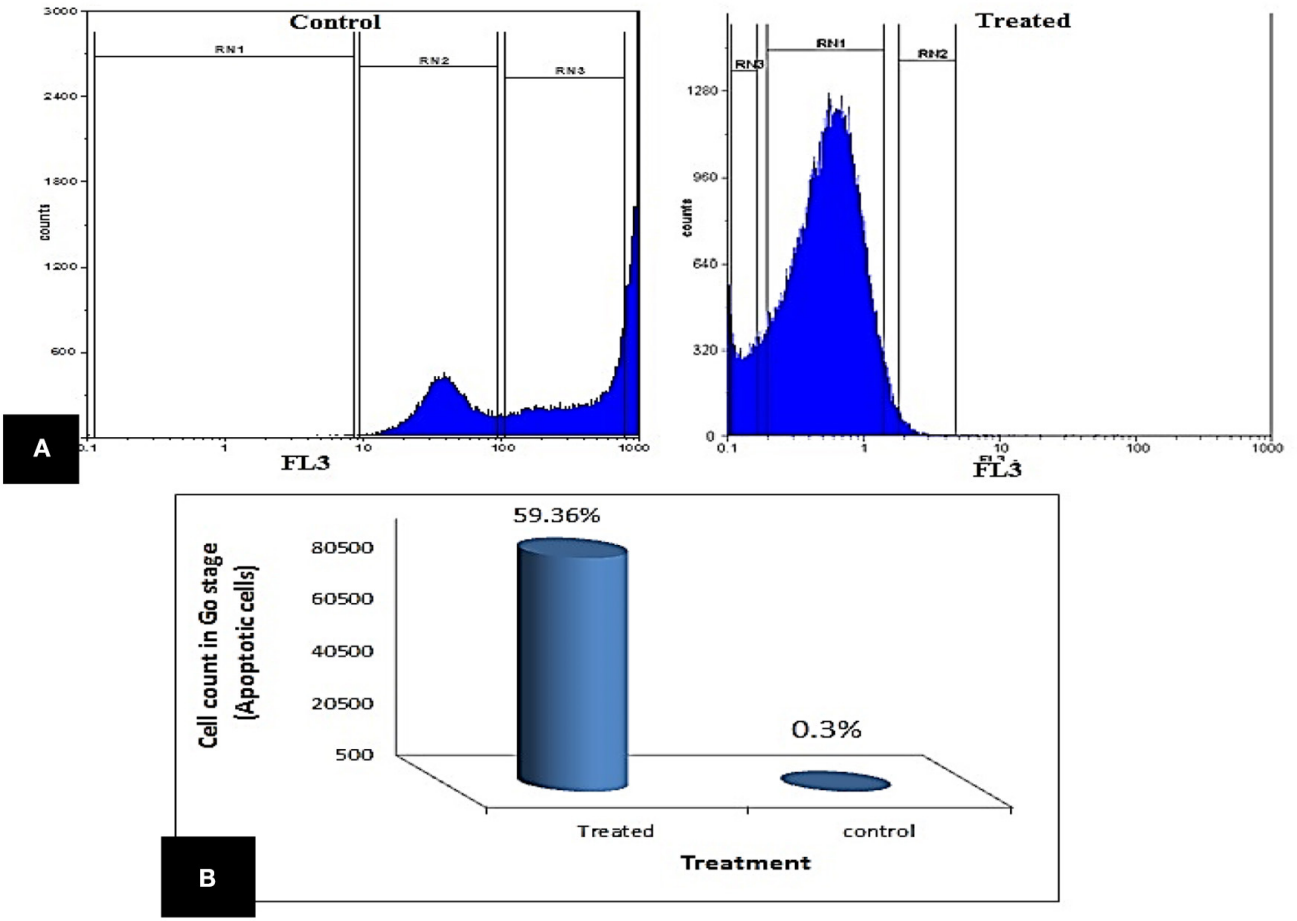
Cell cycle of HepG2 showing **(A)** the ability of enterocin LNS18 to arrest the cycle at G0 causing apoptosis, **(B)** the percentage of treated cells (59.36) arrested at G0.

The impact of enterocin LNS18 on HepG2 cell cycle distributions was measured by flowcytometry. It revealed a significant increase in the cell count at G0 phase (59.36% of the treated cells) compared to the control (0.3%). The accumulation of cells in sub G0 cells indicated an increase in apoptosis (Figure 4).

**Figure 5:**
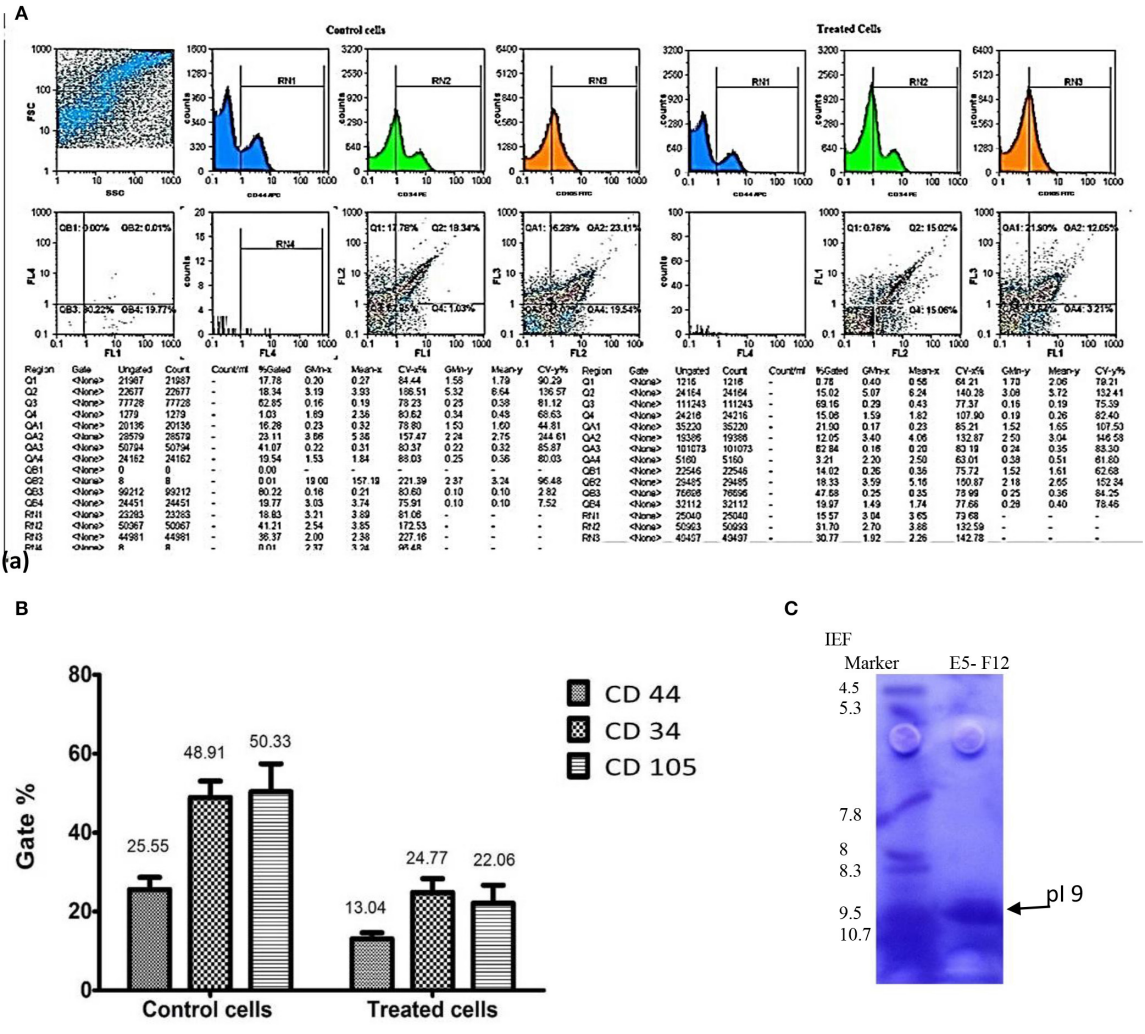
Flow cytometry output of HepG2 CD markers showing dot plots of control and treated cells **(A)**, the percentages of inhibited expression of HepG2 CD markers **(B)**, and electrophoregram showing pI value of purified enterocin LNS18 peptide as pointed by the arrow **(C)**.

Figure 5A represented the effect of enterocin LNS18 on the expression of HepG2 CD markers. It showed dramatic reduction in the expression of all CD markers used as recorded in Figure 5B.

The biochemical nature of the test bacteriocin was also assessed. It was found that keeping enterocin LNS18 at room temperature for 30 min resulted in a non-significant decrease in the residual antimicrobial activity (96.5%) and its storage at 4°C for 30 min showed no change in the activity. However, a temperature-dependent decrease in the activity was observed at 40 and 60°C where the residual activities were 80.8 and 78.1, respectively. However, when the test bacteriocin was subjected to different pH values, a significant decrease in the activity was observed at pH ≥9, while the total stability was kept at pH 2–8 (Supplementary Data 3). Significant reduction in the activity occurred at higher temperature (100°C and above) while non-significant reduction was observed following enzymatic digestion by proteinase K. Additionally, there was a marked reduction in the activity after treatment with trypsin and α-chymotrypsin (32.2 ± 0.001 and 41.7 ± 0.02), respectively as shown in Supplementary Data 3. Slight and non-significant decrease in the bacteriocin activity was observed when treated with different detergents and other materials like chloroform.

Isoelectric focusing experiment revealed that the purified bacteriocin was a single protein band with pI value of 9 as shown in Figure 5C.

**Figure 6:**
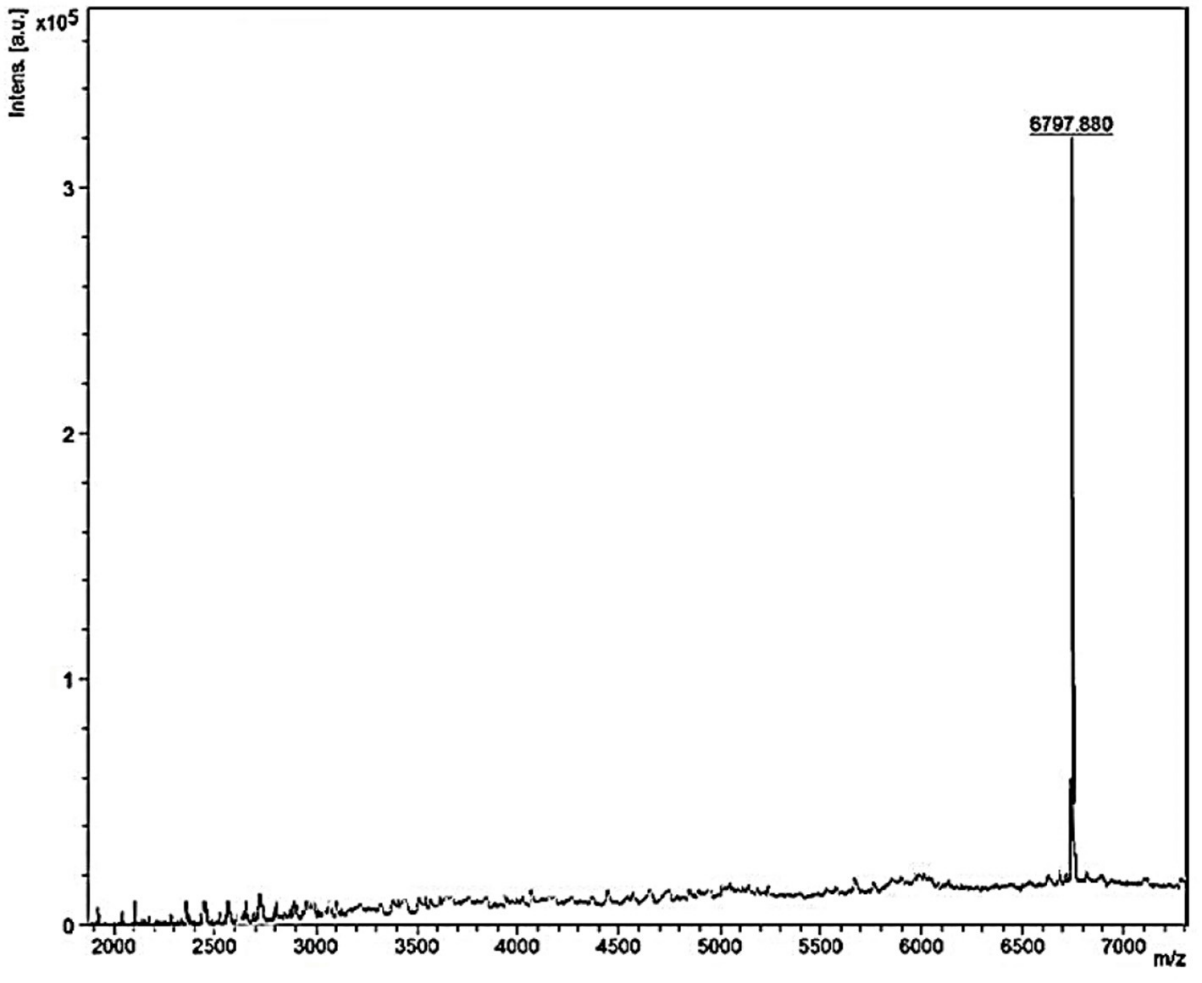
MALDI-TOF MS analysis of the purified and most biologically active bacteriocin (enterocin LNS18) showing a molecular mass of 6797.880 Da.

Mass spectrometry of the purified bacteriocin revealed a single intense peak of a peptide with a molecular mass of 6797.880 Da as detected from Figure 6. Moreover, the predicted pI/MW of LNS18 deduced sequence performed using Compute pI/MW tool: EXPASY was 9.7/8633.32 Da.

Direct Edman degradation sequencing of the test bacteriocin failed initially to give sequences, perhaps due to modified or blocked *N*-terminal amino acid residue, which is normal for circular bacteriocins. Furthermore, when the bacteriocin was subjected to treatment with BNPS-skatole, a reagent that cleaves peptides specifically for the carboxyl side of tryptophan residues, about 415 Da increase in molecular mass was noticed, because of hydrolysis, bromination as well as oxidation reactions. The situation is different for a linear peptide where treatment of it with a cleavage reagent generally yields fragments of lower molecular mass than that of the native peptide. The above-mentioned increase in molecular mass indicated that the test bacteriocin had a circular structure probably due to the presence of a linkage from head-to-tail of both *N*- and *C*-termini. Furthermore, following Edman degradation sequencing of the peptide fragment resulted from BNPS-skatole treatment revealed amino acid sequences of total sixty one residues.

**Figure 7:**
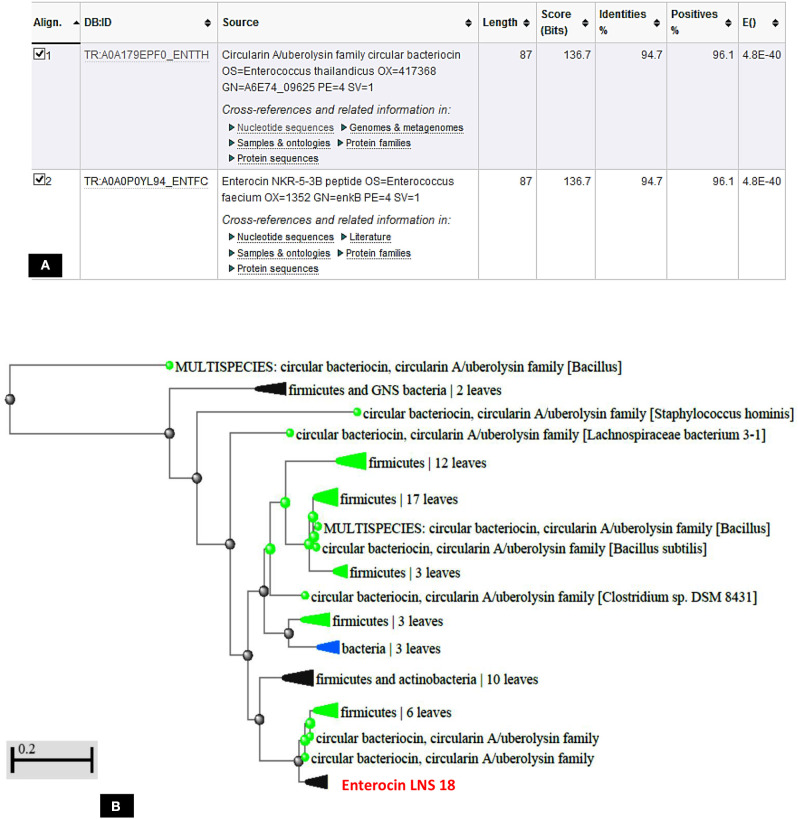
Hit table of LNS18 as a query with the subjects stored in the UniProt Knowledge database showing 94.7% identity with circularin A bacteriocin as well as enterocin NKR-5-3B **(A)**, phylogenetic relationship of LNS18 amino acid sequence from *E. thailandicus* with the subjects of database **(B)**. The scale indicates the evolutionary distance of 0.2 per site.

The deduced amino acid sequence from the nucleotide sequencing of the structural gene of the target bacteriocin showed 86 amino acids as a putative precursor of the bacteriocin with a 22 amino acids forming the *N*-terminal leader. Upon testing the homology of the peptide sequence translated from the putative bacteriocin structural gene with those from UniProt KB, it shared 94.7% identity with circularin A from *E. thailandicus* (A0A179EPF0_ENTTH) as well as enterocin NKR-5-3B from *E. faecium* (A0A0P0YL94_ENTFC) as recorded in the exported hit table shown in Figure 7A. Moreover, a phylogenetic tree presented in Figure 7B showed that the target bacteriocin LNS18 was closer to circular bacteriocin; circularin A/uberolysin family.

**Figure 8:**
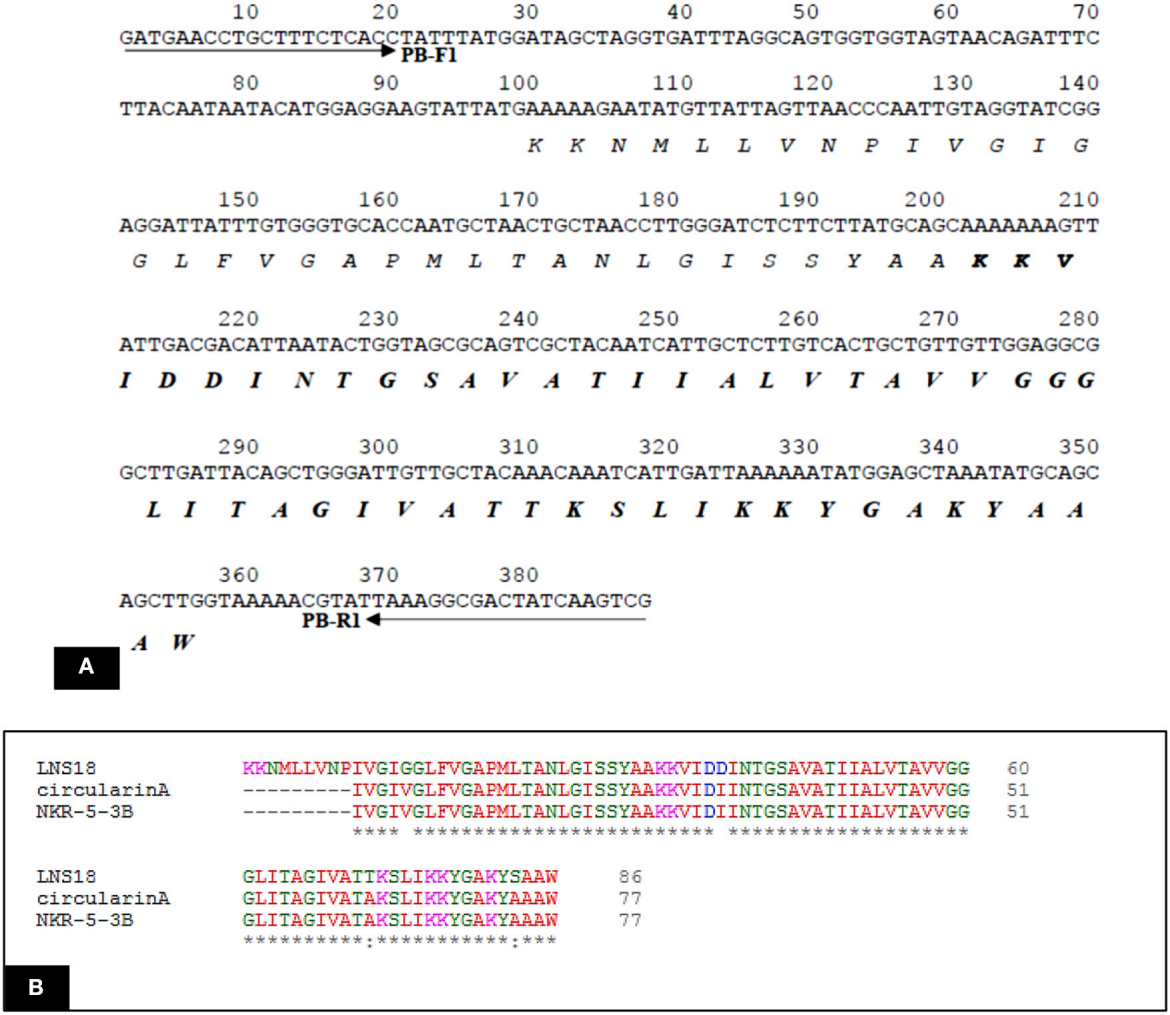
**(A)** Nucleotide sequence of the region encoding bacteriocin (LNS18) structural gene. Solid arrows indicate specific primers. The deduced amino acid sequence is present below the nucleotide sequence and the sequence corresponding to the mature LNS18 peptide is indicated in bold. **(B)** Multiple sequence alignments of Query LNS18 peptide with circularin A bacteriocin produced by *E. thailandicus* (A0A179EPF0_ENTTH) and in comparison with enterocin NKR-5-3B from *E. faecium* (A0A0P0YL94_ENTFC). Conserved residues were presented as (*) which means identical residue and (:) means conserved substitution. Multiple alignments were generated using CLUSTAL omega program with its default parameter setting (http://www.ebi.ac.uk/Tools/msa/clustalomega/).

**Figure 9:**
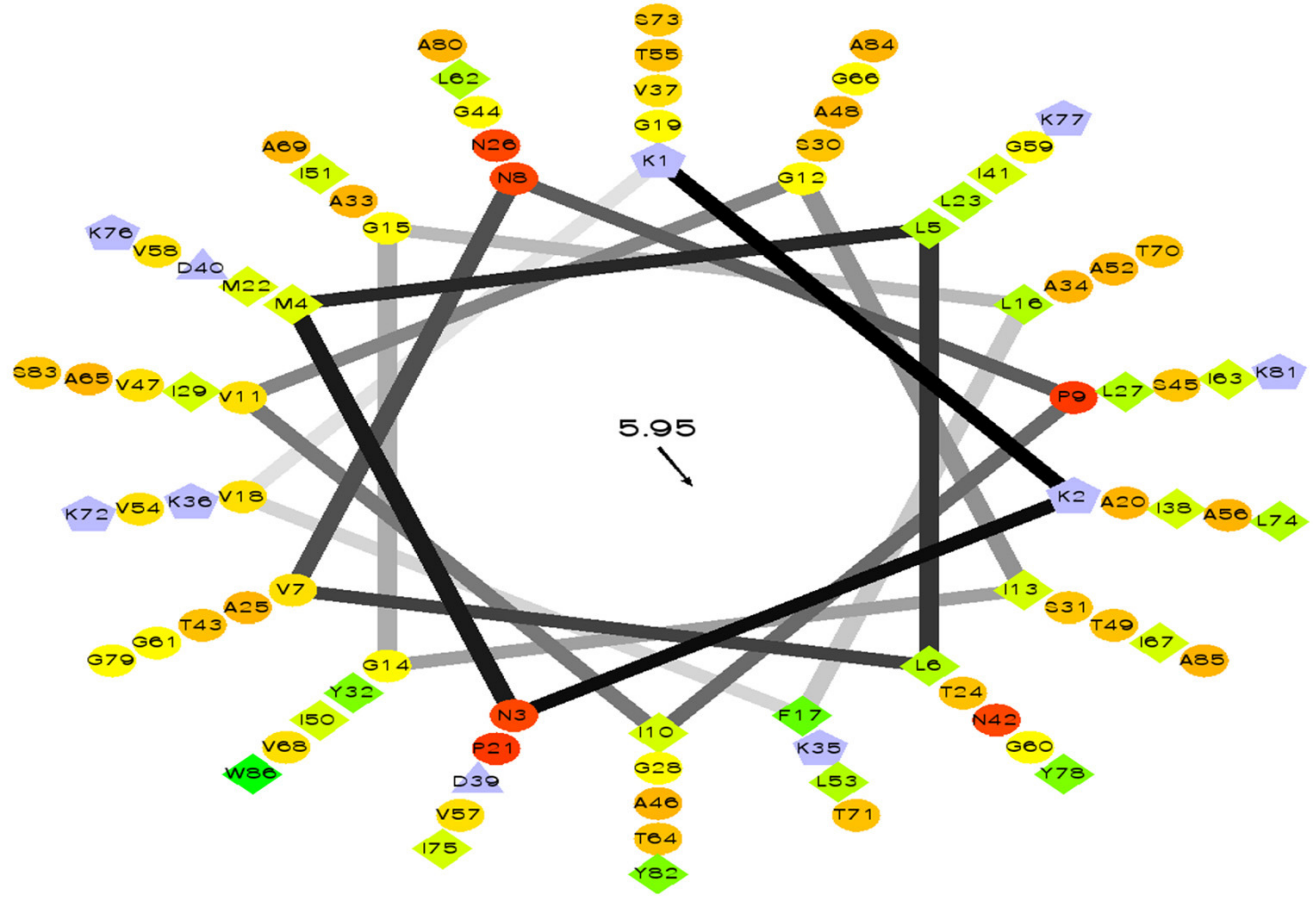
Helical wheel output of LNS 18 bacteriocin obtained from *E. thailandicus*, showing hydrophobicity °, hydrophobic residues as >, potentially negatively charged as Δ, and potentially positively charged as Â. Hydrophobicity is color coded as well: the most hydrophobic residue is green, and the amount of green is decreasing proportionally to the hydrophobicity, with zero hydrophobicity coded as yellow. Hydrophilic residues are coded red with pure red being the most hydrophilic (uncharged) residue, and the amount of red decreasing proportionally to the hydrophilicity. The potentially charged residues are light blue. http://rzlab.ucr.edu/scripts/wheel. Id: wheel.pl,v 1.4 2009-10-20.

Query amino acid sequence of LNS18 was subjected to multiple sequence alignments using Clustal Omega with circularin A from *E. thailandicus* (A0A179EPF0_ENTTH) as well as enterocin NKR-5-3B from *E. faecium* (A0A0P0YL94_ENTFC). It revealed 73 conserved identical residues and 2 conserved substitutions including; Ile71 and Ser83 instead of Ala 71 and Ala 83, respectively. Additionally, nucleotide sequence of the region encoding bacteriocin (LNS18) structural gene as well as the deduced amino acid residues were presented in Figure 8. Moreover, the properties of the amino acids forming LNS18 bacteriocin presented as helical wheel (Figure 9).

**Figure 10:**
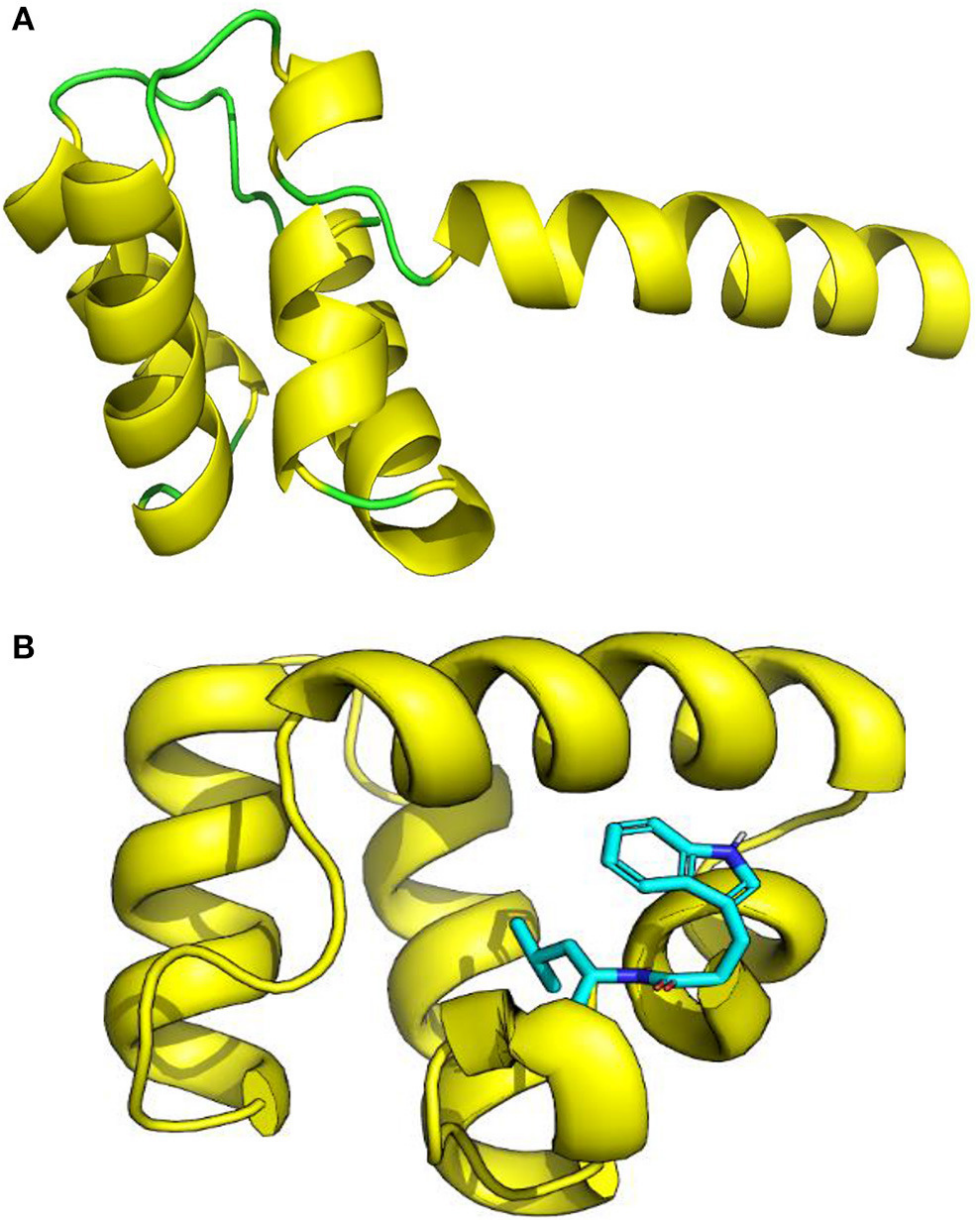
3D Model of enterocin LNS18 presented as cartoon showing **(A)** yellow α-helices and green loops, **(B)** a predicted 3D homology modeling of enterocin LNS18 after release of 22 amino acids and head to tail circularization between tryptophan and leucine amino acid residues. Images were generated by PyMol (1.8.3, Schrödinger, LLC.).

**Figure 11:**
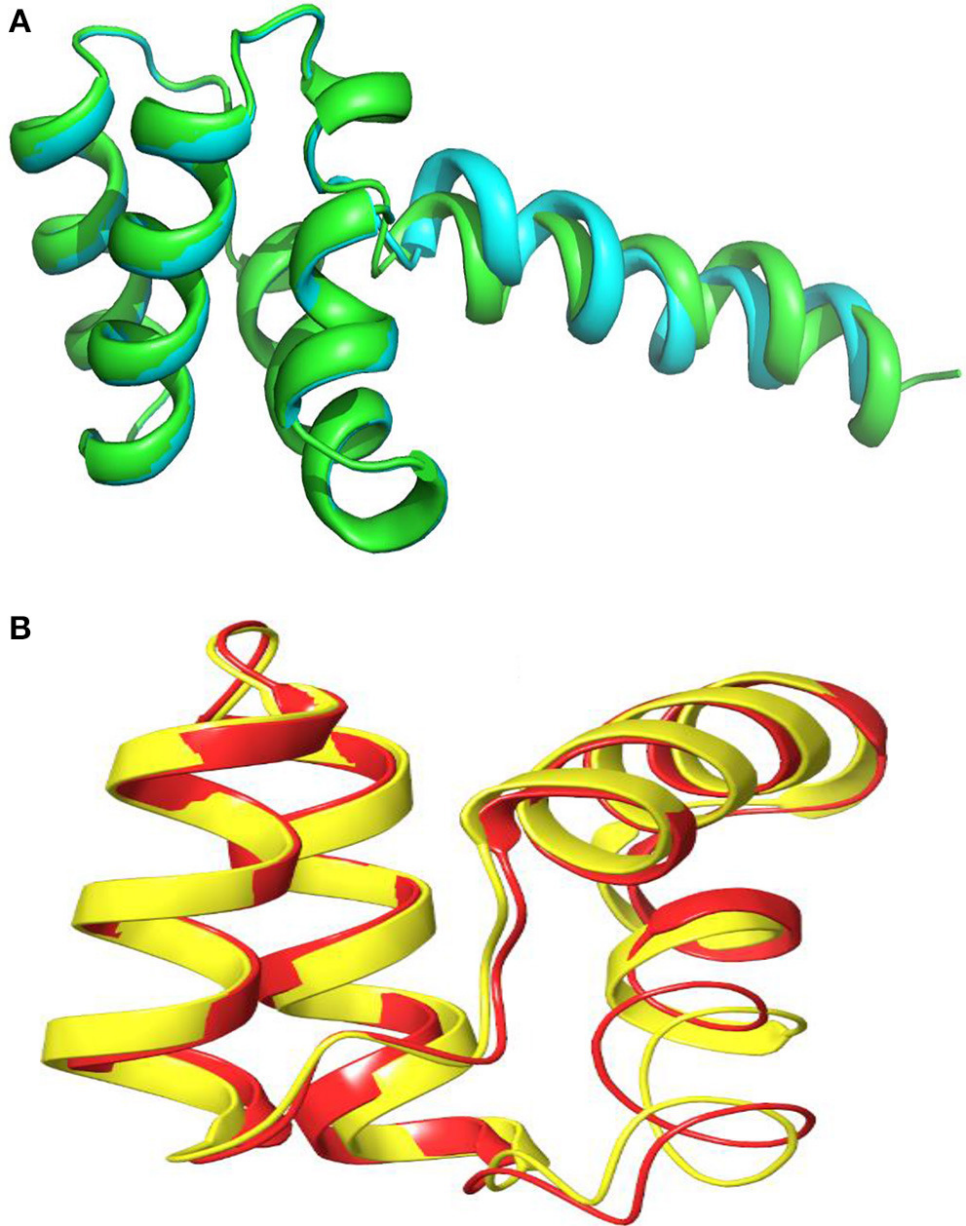
Alignment of 3D-Model of enterocin LNS18 and enterocin NKR-5-3B bacteriocin produced by *E. faecium* (A0A0POYL94_ENTFC) showing that **(A)** both of them (LNS18 in cyan and NKR-5-3B in green) share most of the deduced amino acid structure. **(B)** The circular active parts of LNS18 (in red) with enterocin NKR-5-3B bacteriocin (in yellow) are superimposed. Cartoon model images were generated by PyMol (1.8.3, Schrödinger, LLC).

**Figure 12:**
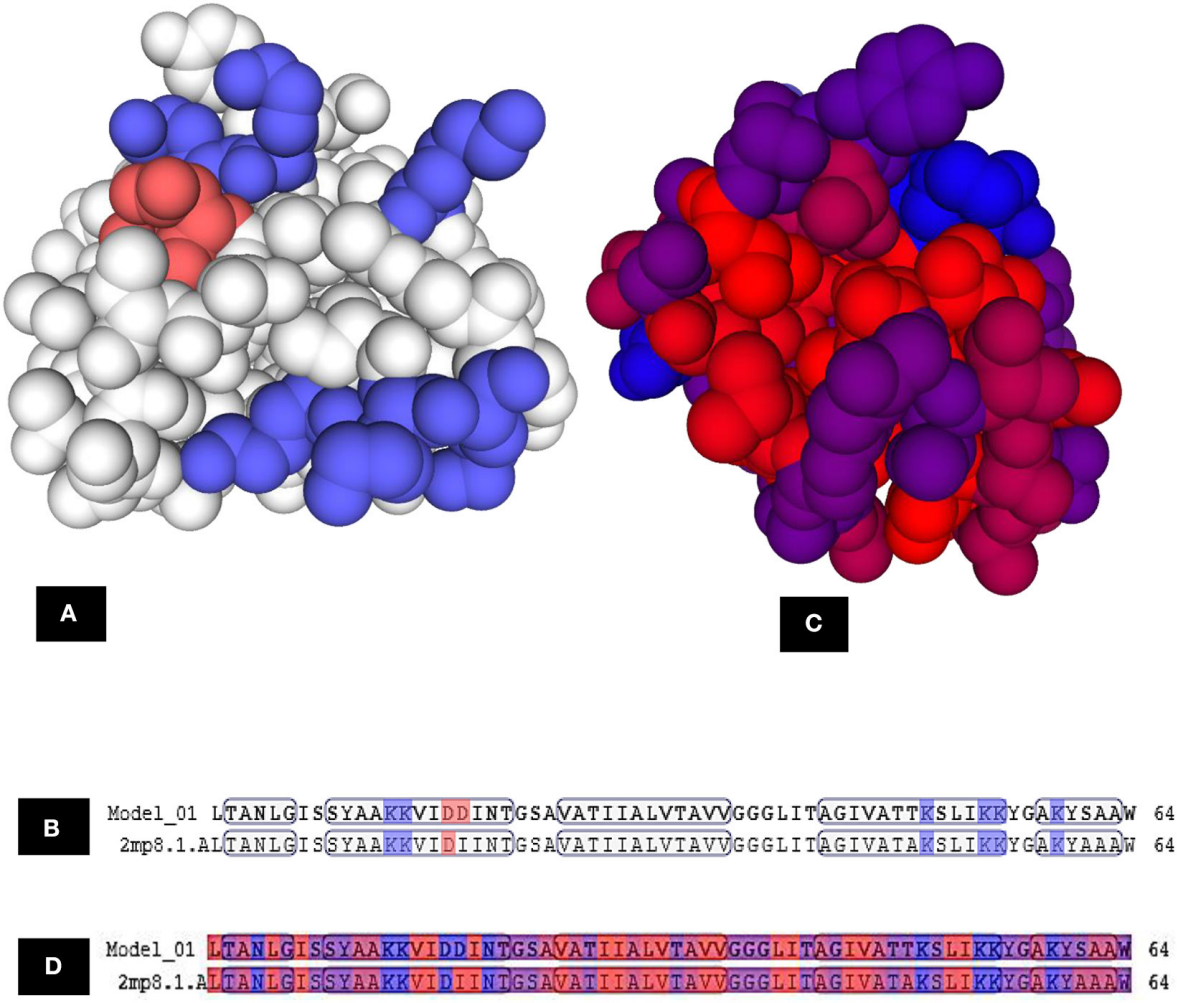
Model–template alignment showing **(A)** the areas of positive charge presented in blue, areas of negative charge in red and the neutral represented in white, **(B)** sequence alignment presenting the amino acids responsible for the charges where repeated lysine (K) provided the structure the positive charge and aspartic acid (D) was responsible for negative charge, **(C)** hydrophobic areas where amino acid residues colored on a spectrum from red (the most hydrophobic) to blue (the most hydrophilic), **(D)** sequence alignment presenting the amino acids responsible for hydrophobicity. Model; LNS18, template; NKR-5-3B. https://swissmodel.expasy.org.

The three-dimensional (3D) model designed as cartoon representation showed six α-helices; five of them appeared in a circular form (not completely closed) and the remaining was linear (Figure 10A). The predicted 3D homology model of LNS18, after head to tail circularization and release of 22 amino acids, showed the formation of a bond between tryptophan (Trp86) and leucine (Leu23) amino acid residues at the site of circularization (Figure 10B). Enterocin NKR-5-3B from *E. faecium* (A0A0P0YL94_ENTFC) was used as a template as it has 3D NMR solution structure. The model LNS18 shared 95.31% identity with the template NKR-5-3B. Model-template alignment showed homology with the circular as well as the linear parts (Figure 11). Furthermore, model–template alignment presented in Figures 12A,B showed areas of positive charges presented in blue due to the presence of 6 lysine amino acid residues resulting in a net positive charge of +4. Hydrophobicity of the structure was also evaluated showing high percentage (55%), mostly due to the presence of Ala (18.75%), Ile (12.5%), Val (10.94%), and Leu (7.81%) as calculated by Prot pi (https://www.protpi.ch/Calculator/ProteinTool/) and presented in Figures 12C,D.

**Figure 13:**
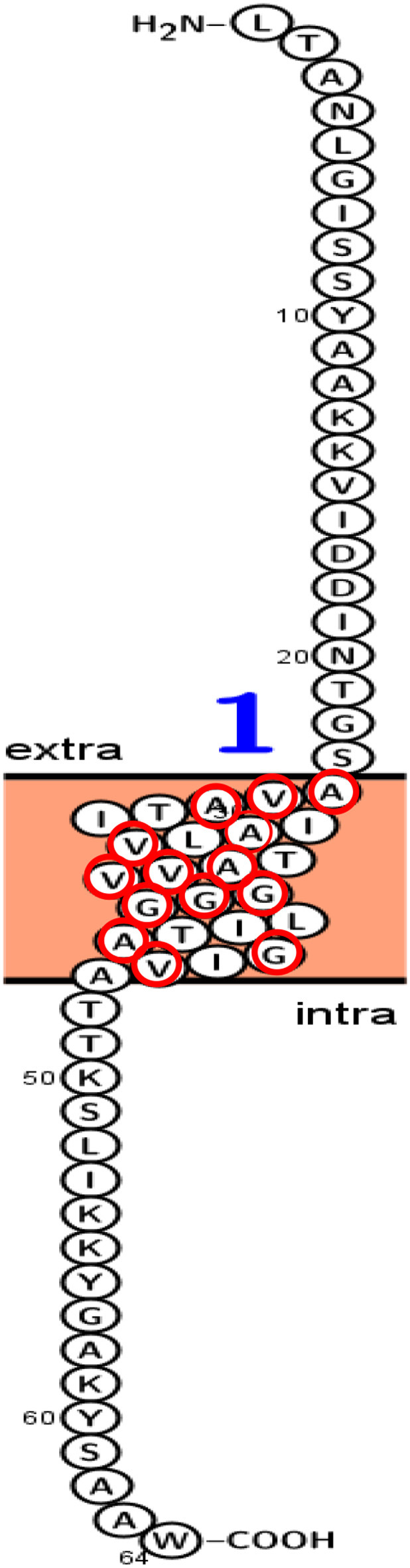
Predicted topology of the membrane enterocin LNS18 showing 1–23 amino acid residues outside the membrane, one transmembrane helix (24–46) with total entropy model of 17.0045 and entropy of best path 17.0050. Glycine (G), valine (V) and alanine (A) amino acids appeared transmembrane localized and they were surrounded with red circles. This figure was designed by Protter (http://wlab.ethz.ch/protter). Data generated by http://www.enzim.hu/hmmtop/html/submit.html (Tusnády and Simon, 1998, 2001).

Figure 13 presented the predicted topology of the membrane LNS18 bacteriocin showing one transmembrane helix (24–46) with no signal peptides or *N*-glyco motifs, as designed by Protter (http://wlab.ethz.ch/protter). Alanine, glycine, valine were observed to be transmembrane localized common amino acids as seen in Figure 13.

## Discussion

In the present work, the production of a broad-spectrum bacteriocin from *E. thailandicus* (E5), isolated from dairy products and identified at the molecular level, was confirmed as recorded in the results section. Bacteriocins are known to have different valuable biological activities as reported in the literature including; treatment of pathogen-associated diseases, cancer therapy, food biopreservation, antiviral activity due to interference with the late stages of virus replication, and acting synergistically with bacteriophages against different pathogens (Yang et al., 2014; Chikindas et al., 2018; Hammami et al., 2019; Meade et al., 2020). Therefore, our aim was to find a bacteriocin with promising antineoplastic activity particularly against hepatocellular carcinoma because it is a major health problem and a third leading cause of death worldwide (Asghar and Meyer, 2012). Interestingly, the purified enterocin LNS18 presented the highest cytotoxicity percentage (75.24) against HepG2 tumor cells *in vitro* at 100 μM while only 7.3% of the normal fibroblast were suppressed indicating greater selectivity toward cancer cells. Chumchalova and Smarda (2003) and Villarante et al. (2011) reported that pore-forming colicin A & E1 (from *E. coli*), and pediocin PA-1 (*Pediococcus acidilactici* K2a2-3) inhibited 11 types of cancer cell lines by 17–40%. Moreover, the *in vitro* cytotoxic assays give useful information to define the intrinsic ability of molecules to exert cell death or even loss of viability as a consequence of destruction to several cellular functions (Paiva et al., 2012). Furthermore, enterocin LNS18 was potent (IC50 = 15.643 μM) against HepG2 cells compared to bacteriocins reported in the literature. Furthermore, the calculated selectivity index was found to be 14 reflecting the greater window between therapeutic and toxic effects. Paiva et al. (2012) reported that the IC_50_ of bovicin HC5 and nisin against HepG2 were 289.30 and 112.25 μM, respectively. Additionally, Hassan et al. (2015) mentioned that Hepcidin (TH1-5) showed cytotoxic activity against breast cancer (MCF7) cells with an IC_50_ of 20 μg/ml. Baindara et al. (2017) stated that a dose-dependent cytotoxic effect was achieved by LS10 bacteriocin. Differences in toxic concentrations of bacteriocin against cancer cells might be based on the mechanism of action. Our study addressed some of the mechanisms on which enterocin LNS18 was dependent on its cytotoxic action against liver cancer cells. Among these mechanisms, induction of intracellular ROS production in HepG2 compared to fibroblasts as well as significant increase in the percentage (59%) of HepG2 cells arrested at G0 compared to the control indicating apoptosis. Furthermore, the expression of HepG2 CD markers was markedly reduced. Hetz et al. (2002) reported that *Klebsiella pneumoniae* RYC492 secreted microcin-E492 which had the ability to induce apoptosis on breast cancer cell lines at 10 μg/ml while higher concentrations could result in necrosis. Alterations in the cell cycle of MCF7 cell line were exerted by colicin E1 that showed an elevated number (26%) of the cells retained at G1 phase (Kaur and Kaur, 2015). Furthermore, Matuszewska et al. (2018) reported that low molecular weight bioactive substance isolated from cultures of wood degrading fungus *Cerrena unicolor* showed antibacterial activity at much lower concentration (6.25 μg/ml) than that showing anticancer activity (300 μg/ml). Similar findings were obtained by enterocin LNS18 (data not shown). Moreover, enterocins are known with their diversity even for those from the same bacterial strain (Franz et al., 2007). For example, Himeno et al. (2015) reported that a strain of *E. faecium* NKR-5-3 produced five bacteriocin peptides, termed as enterocins NKR-5-3A, B, C, D, and Z from different fractions. Enterocins NKR-5-3A and Z belonged to class IIb bacteriocin, Enterocin NKR-5-3C was identified as a novel class IIa with potent anti-listerial effect, Enterocin NKR-5-3D belonged to class IId bacteriocins that have weak antibacterial activity. Similar result was mentioned by Gaaloul et al. (2014). Hence, different bacteriocin peptides produced by a single strain belonged to various classes and showed different biological activities. This might explain the opposite cytotoxic behavior of fractions 8 and 19, compared to others, recorded in our study.

Being proteinaceous in nature, enterocin LNS18 showed a decrease in its antimicrobial activity after treatment with proteinase K but it was non-significant. On the other hand, the use of α-amylase did not result in a change in the bacteriocin activity suggesting that it was not glycosylated. Similarly, the effect of lipase or catalase enzymes reflected the absence of a lipid anchor. Non-significant decrease in the bacteriocin activity was recorded also after lysozyme treatment. Similar data reported by Line et al. (2008). Enterocin LNS18 was thermally stable (up to 70°C) and also presented stability at a wide range of pH (2–8). The study of Himeno et al. (2015) reported that enterocin NKR-3-5B showed stability against thermal and pH stress as well as proteolytic digestion. The study of Line et al. (2008) reported that enterocin-760 was stable at different pH degrees either acidic or alkaline conditions (pH 3–9). It also showed an isoelectric point (IEF) of 4.8. The study of Šeatović et al. (2011) mentioned that bacteriocin G2, synthesized by the *Lactobacillus plantarum* G2, showed pI value of 10, which indicates the cationic nature that creates attraction between the positive charges of bacteriocin and the anionic surface of microbial membranes or cancer cells. In the current investigation, subjection to isoelectric focusing experiment revealed a single protein band with IEF value of 9, which agreed with the calculated one. This cationic peptide is responsible for the exhibited activities discussed above (Kaur and Kaur, 2015). Additionally, enterocin LNS18 showed MW of 6797.880 Da that was accurately determined by MALDI-TOF-MS and it was very close to the calculated one. Himeno et al. (2015) reported similar MW of enterocin NKR-5-3B (6316.4 Da).

Antimicrobial peptides in which *N*-to-*C*- terminal linked covalently forming conserved circular structures are termed circular bacteriocins (Cebrian et al., 2010). The later peptides synthesized as linear precursor with a leader sequence that varies in length and undergoes enzymatic cleavage during maturation. Hence, it goes through three stages: leader sequence splitting, circularization, and release to the outside of the bacterial cell (Gabrielsen et al., 2014). Additionally, the leader peptides consisted of 2–48 amino acid residues while mature circular bacteriocin composed of up to 70 amino acids with molecular weight ranged between 5.6 to 7.2 kDa. Moreover, circular bacteriocins are generally characterized by a broad spectrum of antimicrobial activity, thermal, pH stability and proteolytic resistance and high pIs reached 10 (Gabrielsen et al., 2014; Himeno et al., 2015). In the present study, enterocin LNS18 consisted of 86 amino acids (deduced from nucleotide sequence) indicating that it is immature and it would undergo enzymatic cleavage. Subjecting the sequence to BLASTp for homology, it revealed 94.7% identity with circularin A/uberolysin family of *E. thailandicus* (TR: A0A179EPF0_ENTTH) and also with Enterocin NKR-5-3B from *E. faecium* (TR: A0A0P0YL94_ENTFC). Moreover, enterocin NKR-5-3B composed of 87 amino acid residues and contained a precursor peptide that was processed enzymatically via adjacent cleavage and ligation of Leu24 and Trp87 to give the mature (circular) bacteriocin (Himeno et al., 2015). In the current investigation, the predicted homology 3D model showed complete circularization of enterocin LNS18 structure and a bond formed between both Trp86 and Leu23 amino acids. This was associated with release of the 22 amino acids that were forming the linear portion of the bacteriocin (leader peptide). However, Kemperman et al. (2003) reported that circularin A, synthesized by *Clostridium beijerinckii* ATCC 25752, consisted of a prepeptide of 72 amino acid residues. A circular mature antimicrobial peptide of 69 amino acids was produced due to cleavage of the prepeptide between Leu3 and Val4 residues followed by a head-to-tail ligation of *N-* and *C-*termini.

Construction of LNS18 3D model revealed the presence of five circular α-helices and a single linear one while that of NKR-5-3B consisted of four α-helices although they shared most of the sequence. This was explained by Himeno et al. (2015) who reported that the four α-helices of enterocin NKR-5-3B could superimpose with the five α-helices of enterocin AS-48 where helices 1–3 of enterocin NKR-5-3B superimpose well with those corresponding in enterocin AS-48 and the fourth helix was replaced with two helices (in enterocin AS-48) that could retain the overall fold. Hence, this strongly supports the presence of shared conserved fold in all circular bacteriocins. Moreover, not only the fold but also the charge distribution of enterocin NKR-5-3B and enterocin AS-48 where the former showed a net charge of +5, resulting from the presence of six Lys residues while enterocin AS-48 was more polar as it has eight Lys, four Glu and two Arg residues showed a net charge of +6 (Gonzalez et al., 2000; Martin-Visscher et al., 2009). For LNS18, it had six Lys residues resulting in a net charge of +4. Accordingly, most circular bacteriocins show a high net positive charge and so target the membrane selectively based on the electrostatic interaction.

The process of circularization usually occur within a hydrophobic region where a high steric hindrance is present. This hydrophobicity presented in 55% of LNS18 structure mostly due to the presence of Ala, Ile, Val, and Leu that present in high percentages relative to other residues. Interestingly, it was previously reported that circular bacteriocin family shared a common structural fold, termed saponin fold where helices folded to form a globular domain characterized with a hydrophobic core as well as a hydrophilic surface. For example, the compact hydrophobic core of enterocin NKR-5-3B had primarily contained the hydrophobic side chains of amino acids; Leu, Ile, Ala, and Val. Additionally, it had a highly dynamic and hydrophilic surface loops contained smaller residues of Gly and Ser contributing to their flexibility. Moreover, such loops are essential for the folding and cyclization of peptides inducing the helical segments to move toward the position bringing the termini into proximity and hence the cyclization event occurs (Cebrian et al., 2010; Van Belkum et al., 2011; Gabrielsen et al., 2014; Himeno et al., 2015; Dreyer et al., 2019). Interestingly, many research articles have reported the greatest importance of the biological activities of circular peptides as antitumor drugs. LAK and Phakellistatin 2 were examples of cationic peptides that could induce tumor cytotoxicity as they were strongly attracted toward the negatively charged surfaces of the phospholipid bilayer membranes (Martin-Visscher et al., 2009; Blanco-Míguez et al., 2016). In the present investigation, membrane topology of enterocin LNS18 revealed that 23 amino acids of the *N*-terminal part of the peptide sequence interacted extracellularly enabling the one α-helix with of the *C*-terminal part to be transmembrane-localized. This helix was rich in glycine, valine and alanine suggesting high cell death activity. Lohans and Vederas (2012), mentioned that the activity of class IIa bacteriocins such as leucocin A and carnobacteriocin BM1 could be enhanced by replacement with amino acid residues such as glycine, valine, and alanine. Furthermore, Tymoszewska et al. (2017) reported that an interaction occurred between α-helices with proline and alanine residues predicted to be transmembrane-localized assuming to be a part of an inner pore helix. This type of receptor interaction might trigger structural changes inducing permease to open as a pore leading to cytoplasmic leakage and cell death.

In conclusion, enterocin LNS18 was a purified bacteriocin produced by *E. thilandicus* isolated from dairy products. It showed a broad spectrum of antibacterial activity. Its biochemical nature was characterized by subjection to different treatments that reveled thermostable peptide that kept its activity at pH range of 2–8. It showed *in vitro* selective cytotoxicity against HepG2 cells at micromolar concentrations with an IC_50_ of 15.643 μM. It was able to induce ROS, decrease the expression of HepG2 CD markers and arrest the cell cycle at G0. The structure elucidation showed the presence of a circular bacteriocin containing lysine amino acids providing the net positive charge on the structure. The circularization point was predicted between leucine and tryptophan amino acids. Accordingly, these data provide new insights into a promising, selective and biologically active bacteriocin against liver cancer.

## Data Availability Statement

The datasets generated for this study can be found in the an accession number of MF973085 (https://www.ncbi.nlm.nih.gov/nuccore/MF973085.1/).

## Author Contributions

LA-M, NE-D, AR, and AKa conceived the experiments. LA-M, NE-D, AKa, AR, MN, and AMK conducted the experiments and analyzed the results. All authors wrote and reviewed the manuscript.

## Conflict of Interest

The authors declare that the research was conducted in the absence of any commercial or financial relationships that could be construed as a potential conflict of interest.
