# Supplementary material for: Purification, Characterization, Identification, and Anticancer Activity of a Circular Bacteriocin From Enterococcus thailandicus
Source: Front Bioeng Biotechnol. 2020 Jun 23;8:450. doi: 10.3389/fbioe.2020.00450 (PMC7324803; doi:10.3389/fbioe.2020.00450)
Supplement: Supplementary file 1 [file Data_Sheet_1.docx]

| **Indicator**  **strains**  **CFS from *Enterococcu***  **isolates** | **Inhibition zone diameters (mm) formed against indicator strains after treatment of CFS from enterococcal isolates with** | | | | | | | | | | | |
| --- | --- | --- | --- | --- | --- | --- | --- | --- | --- | --- | --- | --- |
|  | 1N NaOH (Neutralization) | | | | Catalase | | | | Proteinase K | | | |
|  | ***S. aureus*** | ***St. thermophilus*** | ***E. coli*** | ***Ps. aeruginosa*** | ***S. aureus*** | ***St. thermophilus*** | ***E. coli*** | ***Ps. aeruginosa*** | ***S. aureus*** | ***St. thermophilus*** | ***E. coli*** | ***Ps. aeruginosa*** |
| **E_5 -_CFS** | 26±0.17 | 25±0.23 | 14±0.04 | 15±0.35 | 26±0.14 | 25±0.02 | 14±0.01 | 15±0.25 | 20±0.07 | 21±0.13 | 10±0.32 | 8±0.15 |
| **E_9-_CFS** | 25±0.44 | 27±0.31 | 10±0.25 | 10±0.24 | 0 | 0 | 0 | 0 | 29±0.14 | 27±0.11 | 12±0.20 | 11±0.17 |
| **E_12-_CFS** | 19±0.45 | 23±0.06 | 12±0.05 | 11±0.01 | 0 | 0 | 0 | 0 | 23±0.05 | 25±0.07 | 14±0.15 | 13±0.11 |

**Supplementary data 1:** Testing for the potentiality of bacteriocin-producing *Enterococcus* isolates

(a)

(b)

**Supplementary data 2:** Identification of the most promising isolate as well as its bacteriocin kinetics. (a) Phylogenetic tree of *Enterococcus thailandicus* (E5) isolate showing the position of the target isolate (as pointed by the arrow) among the selected *Enterococci* based on 16Sr RNA sequences from NCBI, (b) Growth curve of *Enterococcus* E5 along with the bacteriocin activity over 72 hours.

| **Treatment** | **Bacteriocin residual activity (%±SD)** | ***P*-value** |
| --- | --- | --- |
| **Temperature**:  4 ^o^C, 30 min  25 ^o^C, 30 min  40 ^o^C, 30 min  60 ^o^C, 30 min  70 ^o^C, 30 min  100 ^o^C, 30 min  121 ^o^C, 30 min | 100±0.2  96.5±0.51  80.8±0.33  78.1±0.02  75±0.8  66.5±0.31  51.01±0.2 | 0.04  0.4  0.08  0.08  0.07  0.05  0.03 |
| **pH value:**  2  3  4  5  6  7  8  9  10 | 72.3±0.15  77.6±0.33  83±0.18  98.1±0.5  100.3±0.74  100±0.08  70.6±0.34  39.22±0.2  0±0 | 0.08  0.08  0.07  0.11  0.21  0.13  0.09  0.004  0.000 |
| **Enzymes:**  Proteinase-K  α-amylase  α-chymotrypsin  trypsin  lysozyme  catalase  lipase | 82±0.02  100.4±0.12  32.2±0.06  41.7±0.44  92±0.03  100.1±0.6  100±0.17 | 0.07  0.08  0.02  0.001  0.07  0.06  0.23 |
| **Detergents and others:**  SDS  Urea  Triton X-100  Tween 80  EDTA  Chloroform | 93.3±0.22  95.2±0.05  96±0.45  97.6±0.04  96.4±0.33  91.1±0.08 | 0. 1  0. 43  0.09  0.54  0.07  0.08 |

**Supplementary data 3:** Effects of different treatments on the antibacterial activity of the purified bacteriocin
